# A hybrid BAC physical map of potato: a framework for sequencing a heterozygous genome

**DOI:** 10.1186/1471-2164-12-594

**Published:** 2011-12-05

**Authors:** Jan M de Boer, Theo JA Borm, Taco Jesse, Bart Brugmans, Xiaomin Tang, Glenn J Bryan, Jaap Bakker, Herman J van Eck, Richard GF Visser

**Affiliations:** 1 Wageningen UR Plant Breeding, Wageningen University and Research Centre, Droevendaalstesteeg 1, 6708 PD Wageningen, The Netherlands; 2 KeyGene N.V., P.O. Box 216, 6700 Wageningen, The Netherlands; 3 The James Hutton Institute, Invergowrie, Dundee DD2 5DA, UK; 4 Laboratory of Nematology, Wageningen University, P.O. Box 8123, Wageningen, The Netherlands; 5 The Centre for BioSystems Genomics, Wageningen, The Netherlands; 6 Current address: Rijk Zwaan, Fijnaart, The Netherlands; 7 Current address: Monsanto Vegetable Seeds, Wageningen, The Netherlands; 8 Current address: Department of Biology, Colorado State University, Fort Collins, USA

## Abstract

**Background:**

Potato is the world's third most important food crop, yet cultivar improvement and genomic research in general remain difficult because of the heterozygous and tetraploid nature of its genome. The development of physical map resources that can facilitate genomic analyses in potato has so far been very limited. Here we present the methods of construction and the general statistics of the first two genome-wide BAC physical maps of potato, which were made from the heterozygous diploid clone RH89-039-16 (RH).

**Results:**

First, a gel electrophoresis-based physical map was made by AFLP fingerprinting of 64478 BAC clones, which were aligned into 4150 contigs with an estimated total length of 1361 Mb. Screening of BAC pools, followed by the KeyMaps *in silico *anchoring procedure, identified 1725 AFLP markers in the physical map, and 1252 BAC contigs were anchored the ultradense potato genetic map. A second, sequence-tag-based physical map was constructed from 65919 whole genome profiling (WGP) BAC fingerprints and these were aligned into 3601 BAC contigs spanning 1396 Mb. The 39733 BAC clones that overlap between both physical maps provided anchors to 1127 contigs in the WGP physical map, and reduced the number of contigs to around 2800 in each map separately. Both physical maps were 1.64 times longer than the 850 Mb potato genome. Genome heterozygosity and incomplete merging of BAC contigs are two factors that can explain this map inflation. The contig information of both physical maps was united in a single table that describes hybrid potato physical map.

**Conclusions:**

The AFLP physical map has already been used by the Potato Genome Sequencing Consortium for sequencing 10% of the heterozygous genome of clone RH on a BAC-by-BAC basis. By layering a new WGP physical map on top of the AFLP physical map, a genetically anchored genome-wide framework of 322434 sequence tags has been created. This reference framework can be used for anchoring and ordering of genomic sequences of clone RH (and other potato genotypes), and opens the possibility to finish sequencing of the RH genome in a more efficient way via high throughput next generation approaches.

## Background

The modern cultivated potato (*Solanum tuberosum*) is a heterozygous autotetraploid (2n = 4x = 48) with an estimated haploid genome size of 850 Mb [1]. This polyploid genome configuration reduces the efficiency of potato breeding and makes potato genetics complicated [2]. To circumvent these drawbacks, diploid potato clones (2n = 2x = 24) are often made, which can serve as intermediate steps in a breeding program [3] or can be used as parents for genetic crosses and for mapping of agriculturally important traits [4,5]. However, despite the availability of diploids, the development of physical map resources in potato has until now been very limited. Local BAC maps have been produced for disease resistance gene regions in two diploids and a wild hexaploid species [6-8]. In order to develop a lasting resource for gene identification and map based cloning in potato, we set out to create a genome-wide BAC-based physical map. This physical map is made from the diploid clone RH89-039-16 (hereafter referred to as RH), which is the male parent of the ultradense genetic map of potato [9]. First, an AFLP-based physical map was constructed, and more recently a sequence-tag-based physical map was added, so as to take advantage of the developments in next-generation sequencing. By their method of construction, both potato physical maps differ from previous *de novo *physical maps of plant genomes.

Genome-wide physical maps are made by ordering the clones of a genomic BAC library into groups of overlapping BACs called contigs. To this end, a characteristic DNA band pattern, called a fingerprint, is made from the individual BACs clones, after which they can be ordered into contigs on the basis of similarity in their fingerprint patterns by specialized software like FPC [10]. The published physical maps of plant genomes have shown an evolution of BAC fingerprinting methods over the past decade: starting with agarose gel electrophoresis [11], this was followed by high-resolution sequencing gels [12], which was in turn superseded by multi-colour capillary electrophoresis [13]. All of these fingerprint procedures have relied on restriction enzyme digestion of the BAC DNA. By contrast, the use of AFLP-based BAC fingerprinting is rare, and has so far only been reported for a full genome physical map of a nematode [14] and for a local physical map in potato [7]. The anchoring of physical maps to genetic maps is most often done with RFLP, SNP, SSR or EST markers [15-20]. BAC anchoring by AFLP markers [21] is much less common, but was applied on a scale of respectively 200, 114 and 149 markers for the sorghum and grape physical maps [12,16,20]. The principle of integrating AFLP marker anchoring with BAC AFLP fingerprinting for local physical map construction was reported under the name "KeyMaps" [22,23], but has so far not been applied to a genome-wide physical map.

A recent addition to the spectrum of AFLP applications [24] is the creation of sequence-based physical maps by a whole genome profiling (WGP) strategy [25]. WGP exploits the ability of AFLP to specifically amplify BAC DNA fragments that have been cut at the EcoRI restriction sites, and by high throughput sequencing collects short sequence reads from these EcoRI sites. Because of their fixed and space-separated positions in the genome sequence, these sequence tags are suitable for physical map construction and at the same time provide a scaffold for anchoring whole genome sequence data. Thus, WGP marks a new development in physical map construction that is in line with the current developments in DNA sequencing technology [26].

For the first potato physical map, it was imperative that it would become integrated with the ultradense AFLP marker genetic map of genotype RH [9]. Therefore, it was decided to fingerprint the BAC clones by non-selective AFLP with the enzyme combination EcoRI/MseI, and to apply the KeyMaps procedure [22,23] for anchoring the BAC contigs to the EcoRI/MseI AFLP markers of the genetic map. As an improvement of the KeyMaps anchoring method, we have used a more efficient set of BAC DNA superpools for genetic marker screening of the BAC library. These superpools followed a random k-sets pooling design [27] that allowed genetic marker localisation within quarter library plate segments, which is four times more accurate than the direct full library plate pool screening used in the original KeyMaps protocol.

To improve the quality of the AFLP physical map, and to expedite the sequencing of the RH genome by next generation technology, a second sequence-tag-based physical map was constructed with whole genome profiling of BAC clones [25]. This WGP physical map includes clones from both the restriction enzyme-based BAC library of the AFLP physical map and a second BAC library, which was prepared by random shearing of genomic DNA [28]. This sheared library was anticipated to close remaining gaps in the physical map.

The AFLP physical map has already been in use for sequencing parts of the RH genome [29] and for constructing cytogenetic maps [30,31]. Preliminary results have been published in an overview paper [32], as part of a local sequence map [33] and in a PhD thesis [34]. Presented here are the full methods of construction and characteristics of both the AFLP physical map and the new WGP physical map and their mutual integration. The relevance of these physical maps to potato genomics research is discussed.

## Results and Discussion

### AFLP fingerprinting and AFLP physical map construction

**Figure 1:**
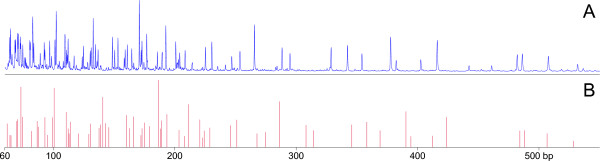
**Example of a non-selective AFLP BAC fingerprint**. **(A) **Original fluorescence trace file from BAC clone RH084E02. **(B) **AFLP band mobilities (in bp) and peak height values extracted from the trace file with band calling software. Only the band mobilities are used for fingerprint alignment by FPC.

The AFLP fingerprinting has been described in detail by Borm [34], and the main results are summarized here. The RHPOTKEY BAC library comprises 78, 336 clones that were obtained from partial digestion of genomic DNA from diploid potato clone RH. All BAC clones were fingerprinted with the non-selective AFLP PCR reaction, using the enzyme combination EcoRI/MseI. The AFLP samples were separated by high throughput capillary electrophoresis in the mobility range 60-900 bp, and AFLP bands were scored from the trace files by band calling software (Figure 1). The number of AFLP bands per BAC followed a bell-shaped distribution, with a peak at 50 bands per BAC. The band density of the AFLP fingerprints was skewed towards the low mobility end of the gels and could be fitted to a truncated geometric distribution. This skewed size distribution has an adverse effect on physical map construction, because it will increase the likelihood of overlaps between unrelated AFLP fragments [35].

**Figure 2:**
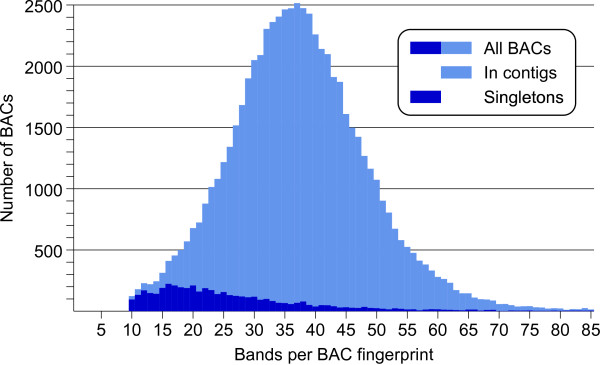
**Distribution of the number of bands per BAC in the 64478 fingerprints of the AFLP physical map**. Counts of BACs in contigs and of singleton BACs are shown separately and are stacked to make the distribution of the complete set of fingerprints.

The final AFLP fingerprint dataset for physical map construction was produced after a number of processing and cleaning steps, involving preliminary versions of the physical map. Removed were chloroplast traces (about 3.8%), artefact band containing traces (about 4.7%), and mixed fingerprints from well-to-well contaminations (about 4.4%). A band size window of 100-650 bp was chosen for fingerprint alignment, and BACs were selected for having between 10 and 100 bands in this interval. Below 100 bp the band density became very high, because of the skewed length distribution of the AFLP fragments. Above 650 bp, many fingerprints were contaminated by size ladder bands due to incomplete separation of the signals from the different fluorescence channels. By applying these two band size cut-offs, the possibilities for false fingerprint alignments are reduced. The result of these filtering operations was a set of 64478 fingerprints with a peak at 37 bands per BAC (Figure 2).

The selection of 64478 AFLP fingerprints, representing 9.6 g.e. of genomic DNA, was aligned into a physical map with the computer program Finger Printed Contigs (FPC) [10]. The BAC contigs were built with a slightly relaxed alignment cut-off value of 1e-09, which maximised the number of incorporated BACs, but also resulted in a high initial number (11221) of questionable fingerprints, with friction alignment. These questionable BAC alignments then were removed for 94.5 percent by re-alignments of the affected contigs with the DQer function at more stringent cut-off values of up to 1e-12. Next, two rounds of automated end-to-end merging were performed on the BAC contigs at more relaxed cut-off values of 1e-08 and then 1e-07. The resulting AFLP physical map has 4150 BAC contigs containing 59747 clones, with an estimated total contig length of 1361 Mb (Table 1; Additional file 1).

**Table 1:** Statistics of the potato AFLP and WGP physical maps

			Contigs per FPC standard size class								BACs										
														
			INF	399	199	99	49	24	9	= 2	per contig		BACs in physical map					Contig length (kb)		Total contig length	
																	
Physical map	Contig count	Contigs	400	200	100	50	25	10	3	BACs	**Avg**.	N50	Total	**g.e**.	Single	Contig	AFLP markers	**Avg**.	N50	Mb	**g.e**.
**AFLP**	All	4150	0	6	23	160	449	1211	1744	557	14.4	25	64478	9.6	4731	59747	1770	306	372	1361	1.60
	> 5 Qs clones *	15	0	2	5	6	2	0	0	0											
	Anchored	1252	0	1	5	90	257	493	373	33	20.4	30						441	542	552	0.65
	WGP-enhanced map, all **	2819	2	14	67	220	396	690	980	450	21.2	46						482	803	1361	1.60
	WGP-enhanced map, anchored **	990	2	8	48	166	249	312	177	28	36.7	59						798	1150	790	0.93

**WGP**	All	3601	0	4	28	121	468	1057	1422	501	14.8	26	65919	9.0	12781	53138	1721	387	559	1396	1.64
	> 5 Qs clones *	76	0	0	7	24	42	3	0	0											
	Anchored	1127	0	1	10	61	287	443	286	39	21.3	30						563	712	634	0.75
	AFLP-enhanced map, all ***	2785	1	12	51	184	391	714	978	454	19.1	40						501	851	1396	1.64
	AFLP-enhanced map, anchored ***	940	1	7	34	132	259	299	178	30	32.7	50						851	1175	800	0.94

* Contigs with more than 5 "questionable" clones (i.e. clones with more than 50% of the bands not aligning within the contig)

** Contig count after incorporation of connections provided by overlapping contigs of the WGP physical map

*** Contig count after incorporation of connections provided by overlapping contigs of the AFLP physical map

### AFLP marker screening of the BAC library

For AFLP marker screening of the RHPOTKEY BAC library, a set of 90 DNA superpools was prepared from 764 quarter library plate pool (QPP) DNA samples, using a random k-sets pooling design [27]. With this pooling design, superpool marker scores are deconvoluted to produce a list of QPPs that contain all the copies of the marker that are present in the superpool set. In this way, marker copies are located in the BAC library within an accuracy of a quarter library plate segment, and this partial marker localization is used as the input information for the *in silico *BAC contig anchoring procedure described in the next paragraph. The characteristics of the BAC pooling design are described in detail in the Methods section.

**Figure 3:**
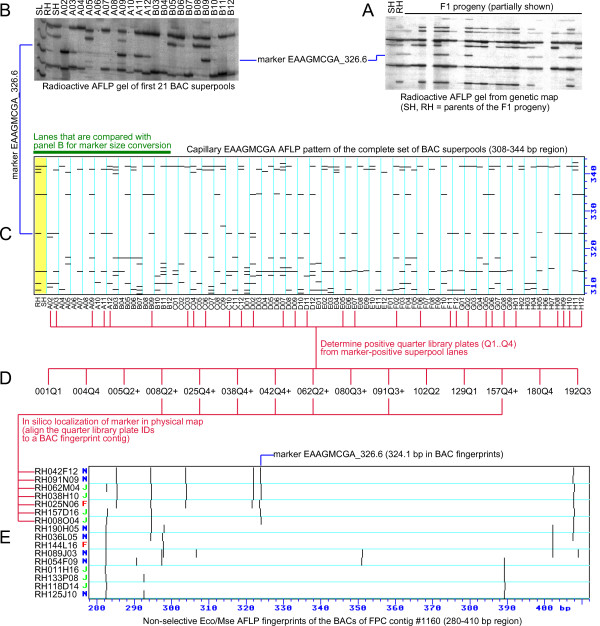
**Flow diagram of the AFLP marker anchoring procedure of the potato AFLP physical map**. The anchoring procedure is illustrated for genetic marker EAAGMCGA_326.6. **(A) **The marker from parent RH is traced back in the original radioactive gel of the genetic map. **(B) **As an intermediate step, a new radioactive gel is made with primer combination EAAGMCGA of the parental DNAs and 21 of the BAC superpools, and the position of the marker is identified. **(C) **The full set of BAC pools is examined with primer combination EAAGMCGA using capillary electrophoresis. The position of the marker is identified in these capillary fingerprints by comparison of the patterns of the first 21 lanes to those in (B). The band scoring interval of marker EAAGMCGA_326.6 was set to 324.0-324.3 bp and the average size was measured to be 324.1 bp. **(D) **Fingerprint bands are scored within this size interval and from the 31 positive superpool lanes (A02....H12) a list of quarter plate pool IDs is generated (001Q1... 192Q3) that are candidates for having the marker. **(E) **Seven of the QPP's identify the contig with the marker in the physical map, on the basis of matching BACs that have the marker band (e.g. BAC RH042F12 is from well F12 of library plate number 042, which is present in quarter plate pool 042Q4).

We have tested 135 selective EcoRI/MseI AFLP primer combinations with 3197 AFLP markers from the RH genetic map [9] on the 90 BAC superpools. These AFLP gels were made by capillary electrophoresis, so that for the anchoring procedure the AFLP marker bands could be directly compared with the AFLP bands in the BAC capillary fingerprints. Figure 3 illustrates the steps of the marker screening procedure.

Because the AFLP markers of the RH genetic map were identified in radioactive gels, it was not possible to locate them directly in the capillary BAC pool gels, since significant shifts in AFLP band mobilities occur between both electrophoresis systems. Therefore, additional radioactive AFLP gels were made of the first 21 BAC superpools and of the parental genotypes of the genetic map. These marker size conversion gels (Figure 3B) formed a bridge between the original radioactive gels of the genetic map (Figure 3A) and the capillary BAC pool gels (Figure 3C), and enabled a reliable marker size conversion between both systems.

For AFLP markers with radioactive sizes below 450 bp, it was found that the shift in band mobilities in the capillary gels varied from 3 bp smaller to 1 bp larger (Additional file 2). Above 450 bp, the capillary sizes were increasingly larger than the radioactive sizes, with up to a +20 bp difference for markers near the maximum (600 bp) of the radioactive size range. The success rate with which the radioactive AFLP markers could be identified in the capillary BAC pool gels was 71%.

### AFLP marker anchoring of BACs

The AFLP marker scores in the BAC superpools were deconvoluted to produce a list of candidate QPPs that may contain the marker (Figure 3D). For markers that had produced an output of at least two QPPs, this list of QPPs was compared *in silico *against the BAC contigs of the AFLP physical map. The physical map location of a marker was determined following the KeyMaps principle, by searching for the BAC contig that had two (or more) overlapping BACs that matched the positive QPPs, and that had an AFLP band in their non-selective EcoRI/MseI fingerprint with the same mobility as the AFLP marker (Figure 3E). Markers with only a single positive QPP could in nearly all cases not be reliably placed on BACs with the *in silico *search, and were omitted from analysis.

**Figure 4:**
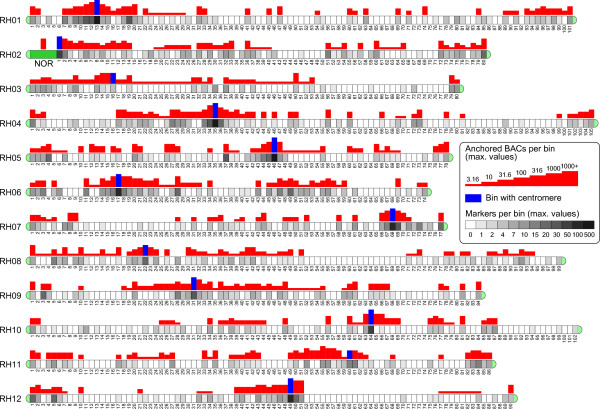
**Genetic map locations of AFLP anchored BACs of the potato AFLP physical map**. The genetic map of parent RH has twelve chromosomes (RH01 to RH012) and is made up from AFLP markers of the enzyme combinations EcoRI/MseI, SacI/MseI and PstI/MseI. Per chromosome, the genetic map is divided into up to 105 numbered bin segments that each represent a distance of one crossover event (0.77 cM) in the mapping population. The number of RH AFLP markers placed in each bin is indicated by a grey intensity value. Red bars indicate the counts per bin of BACs that are anchored to the genetic map by an EcoRI/MseI AFLP marker in their contig. For AFLP markers that mapped to a range of bins, the associated BAC counts have been evenly distributed over these bins. The bins with the centromere have their BAC count shown in blue and follow the identifications by Tang *et al*. [31] and Park *et al*. [42]. The BACs of the Nucleolar Organizer Region (NOR) do not have an AFLP anchor, but were identified by their end sequence. Chromosome orientations are according to bin number in the ultradense genetic map. For alignment to other potato and tomato genetic maps, e.g. from Tanksley *et al*. [43], chromosomes 7, 10 and 12 are in the wrong orientation and must be inverted.

**Figure 5:**
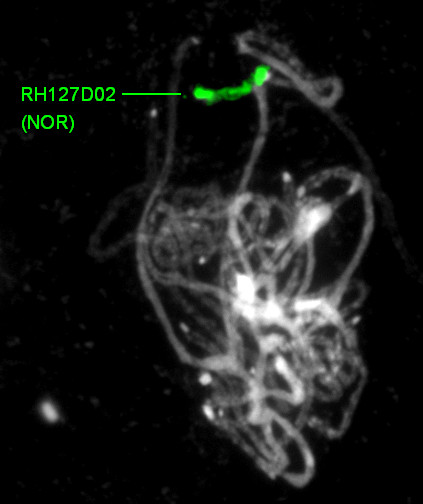
**Identification of the NOR in the AFLP physical map**. Pachytene FISH of BAC clone RH127D02 showed its localisation in the compound structure of the Nucleolar Organizer Region (NOR) on the short arm of chromosome 2 (see Tang *et al*. [31] for methodology). Both brighter fluorescing regions and relatively weaker ones are visible, suggesting differences in NOR chromatin density. In the AFLP physical map the NOR is represented by a 96-clone BAC contig containing RH127D02. In the WGP physical map this NOR contig is absent.

In total 1725 AFLP markers produced an anchor with the *in silico *mapping, placing 1239 contigs containing 25482 BACs on the genetic map (Table 2; Additional file 3; Additional file 4). The distribution of these 25482 anchored BACs across the RH genetic map is shown in Figure 4. Because of local suppression of recombination, the AFLP markers occur in high densities in the centromeric bins of the genetic map, and consequently this is where a large proportion of the anchored contigs are located. Physically, these centromeric bins span a large distance on the cytogenetic map [31], and therefore the genetic distances shown in Figure 4 must be interpreted with caution, because they can give a locally distorted view of distances in the physical map. Chromosomes 3 and 8 innately have relatively few markers in the RH genetic map [9], which is reflected in their low anchoring results. Although the anchored BACs are fairly well distributed on most chromosome maps, genetic regions still exist where anchoring is sparse. For instance, the bin 52-70 region on chromosome 8 is devoid of markers, but was shown to span a significant cytogenetic distance with an estimated length of 5.3 Mb [31]. Also on chromosome 12, AFLP anchoring is sparse in the euchromatic regions of the chromosome arms, especially in the bin 52-90 region, which was recently added to the RH12 map on the basis of FISH analysis [31]. From BAC end sequences, one contig of 96 BACs was identified as having 18S and 26S ribosomal DNA sequences. FISH confirmed that this contig represents the Nucleolar Organizer Region (Figure 5), which is located on the short arm of chromosome 2 in *Solanum *species [36].

**Table 2:** Statistics of *in silico *generated AFLP marker anchors in the AFLP physical map

Chromosome	Markers giving BAC anchors	Anchored BAC contigs	Anchored BACs
RH01	300	211	3720
RH02	120	88	1693
RH03	69	52	1196
RH04	188	139	2882
RH05	202	141	2441
RH06	156	110	2070
RH07	118	94	1755
RH08	69	54	1131
RH09	104	74	1677
RH10	103	73	2347
RH11	137	99	2080
RH12	159	104	2490
Total	1725	1239	25482

The validity of the *in silico*-generated AFLP anchors has been verified by different approaches, such as wet lab testing [34] and *in situ *hybridisation [30,31]. Also, the occurrence of double or triple anchors within contigs of the AFLP physical map (and of the WGP physical map, presented below) has been used to search for invalid anchors. Based on these verifications, 50 *in silico *AFLP anchors were found to be incorrect, which corresponds to an error rate of 2.8 percent across the entire anchoring procedure. The success rate of the *in silico *anchoring step was 76%. When also taking into account the efficiency of the marker size conversion, the overall success rate of BAC anchoring with the 3197 EcoRI/MseI AFLP markers from parent RH was 54%.

The AFLP anchor set of the physical map was extended with 45 AFLP markers that were identified via other routes. Four PstI/MseI markers were identified in BACs by screening a third BAC library of genotype RH. The fingerprints of these positive clones were included in the RH physical map and anchored a large contig to bin 26 of the chromosome 5 genetic map. Local physical map construction in the H1 nematode resistance gene region identified one PstI/MseI and two SacI/MseI markers in the BAC sequences, and anchored contigs to the bin 65 region on chromosome 5 [33]. These additionally anchored contigs are included in Figure 4. More AFLP markers were identified from sequenced BAC clones in the euchromatic regions of chromosome 5, but in most cases these overlapped with the *in silico *AFLP anchors. The AFLP physical map has a total of 7895 BACs in which one or more AFLP markers were identified, and the BAC contigs that are genetically anchored by these seed BACs represent 552 Mb of sequence (Table 1).

### Marker copy number and performance of BAC superpools

**Figure 6:**
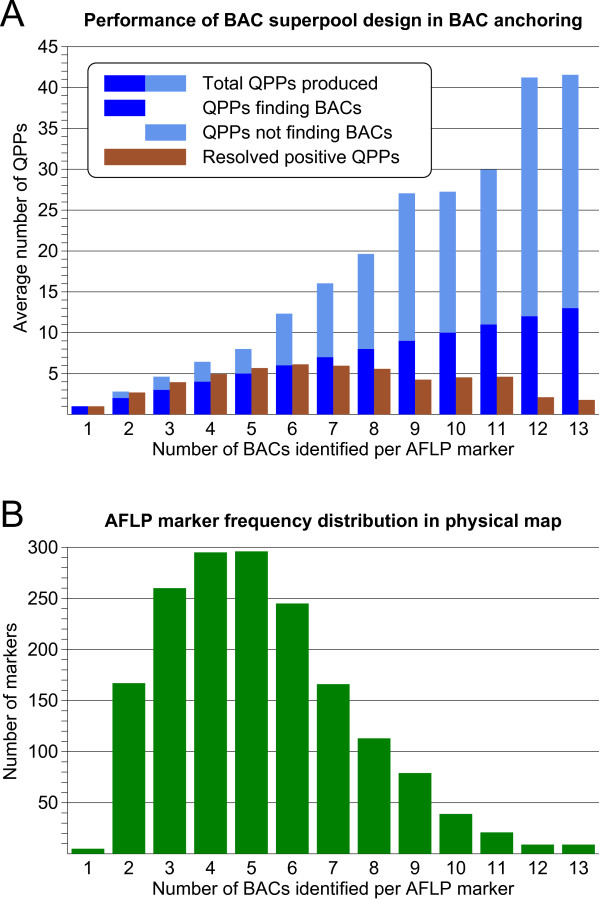
**Performance of the BAC superpool design in AFLP marker anchoring**. **(A) **Numbers of placed (blue) versus unplaced (light blue) QPPs for markers with an increasing BAC copy number in the physical map. The resolved positive QPP counts show the innate capacity of the pooling design to accurately locate markers in the QPPs, which declines at high marker copy numbers. With the AFLP marker anchoring procedure, this decline in pooling design performance was compensated, and markers with relatively high copy numbers were identified on BAC clones without losses. **(B) **Distribution of the number of anchored BACs per AFLP marker. This figure closely represents the marker copy number distribution in the BAC pools.

Figure 6A illustrates how the BAC superpool design has performed in the AFLP marker anchoring procedure. With marker copy numbers of 5 or less, the total number of candidate QPPs produced by deconvolution of the pooling design was close to the number of positive BACs identified for the marker. At higher marker copy numbers, however, an increasing proportion of the candidate QPPs did not find BACs in the physical map. This behaviour was exactly as as predicted from computer simulations with the pooling design (see Methods section), and these unplaced QPPs represent false positives, which begin to appear when the solving capacity of the pooling design is broken down by higher marker copy numbers. For comparison, the intrinsic capacity of the pooling design to recognize true positive QPPs among the QPP deconvolution output is shown with the counts of resolved positive QPPs, which decrease far below the actual marker copy numbers at high marker densities. This condition, however, did not hinder the identification of the marker-positive BACs via the *in silico *anchoring procedure. By linking the deconvolution results of the k-sets pooling design to the KeyMaps anchoring procedure, the performance of the pooling design was enhanced above its intrinsic capacity to resolve the positive QPPs, and full efficiency of marker localisation in the QPPs was retained for the high copy number AFLP markers.

The distribution of the number of BACs identified per marker is shown in Figure 6B. Single copy markers do not contribute to the frequency distribution since they were largely omitted from anchoring. Most AFLP markers had 4 or 5 BACs identified from the BAC superpools. The total amount of BAC DNA represented in the superpools is estimated to be 10 genome equivalents. Since all AFLP markers are, by definition, heterozygously present in the genome, their expected copy number in the BAC pools is 5. Taking into account that slight losses in marker identification will have occurred in the anchoring procedure, our observed average marker count corresponds very well with the expected value for heterozygous markers.

The compact set of 90 BAC superpools, containing 73344 clones, was specifically designed to provide an efficient screening procedure for the heterozygous, and therefore low copy number, AFLP markers in the relatively large 850 Mb potato genome. This screening efficiency was in part achieved by performing the marker localisation only down to the quarter plate pool level. Other marker screening strategies in plant BAC libraries typically have used more than twice the number of BAC pools, while being applied to less clones. For example, in the 750 Mb *Sorghum *genome, a set of 184 six-dimensional BAC library pools containing 24576 clones has been used to locate homozygous AFLP markers on individual BAC clones [12]. The same BAC pooling design has been used for marker screening in 5 g.e. of the heterozygous 475 Mb grape genome [16,20] and with an extension to 208 pools containing 49192 BACs for screening of 6.6. g.e. of the 1115 Mb soybean genome [19]. A drawback of our BAC anchoring procedure, as compared to these other pooling methods, is that single copy AFLP markers cannot be placed on the BAC clones, unless additional wet lab tests are performed.

### Whole genome profiling physical map

**Figure 7:**
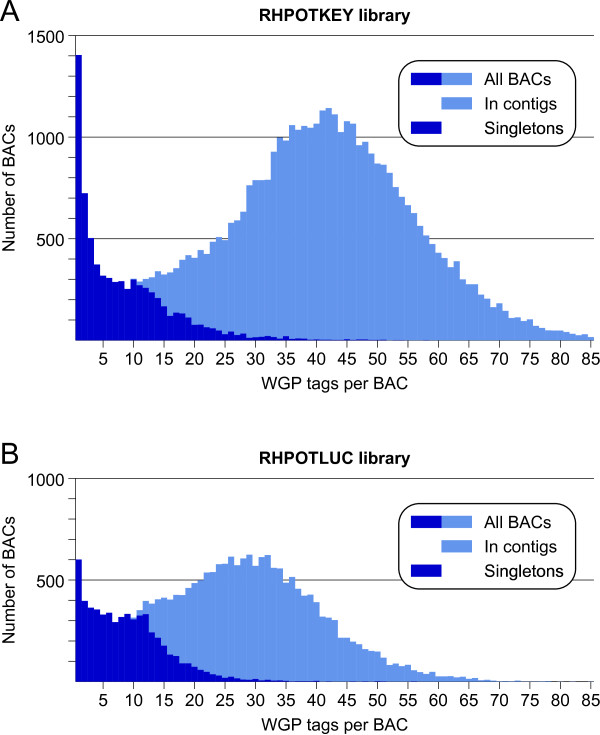
**Distribution of the number of WGP sequence tags per BAC for clones incorporated in the WGP physical map**. **(A) **Distribution for 44292 clones from the RHPOTKEY library. **(B) **Distribution for 21627 clones from the RHPOTLUC library. Counts of BACs in contigs and of singleton BACs are shown separately and are stacked to make the distribution of the complete set of clones.

Whole genome profiling sequence tags were obtained for 44810 clones of the RHPOTKEY BAC library and for 21735 clones of the RHPOTLUC BAC library by high throughput end sequencing of EcoRI/MseI restriction fragments [25]. In total 2248159 sequence tags of 26 bp were assigned to the BAC clones (Table 3). These tags represent 322434 unique sequences, which corresponds to an average distance between tags of 2636 bp on a haploid potato genome length of 850 Mb. The distribution of the number of tags per BAC is shown separately for the two libraries in Figure 7. The RHPOTKEY clones have on average of 38 tags per BAC, whereas the shorter RHPOTLUC clones have on average 26 tags per BAC. Remarkable was that the plates of the sheared RHPOTLUC library systematically had a 20% lower yield of BACs with WGP tags compared to the RHPOTKEY library. This difference most likely is caused by a higher fraction of repeat-rich clones in the sheared library [28], since such clones will fail to resolve their tag sequences with the current WGP sequencing protocol [25].

**Table 3:** Statistics of potato WGP sequence tags

	BAC library		
		
	RHPOTKEY	RHPOTLUC	TOTAL
Library type	partial digest	sheared	
Estimated average clone size (kb)	130	90	
BACs analysed	55296	30720	86016
Genome equivalents analysed *	8.5	3.3	11.7
BACs per sequence run (# runs)	13824 (4)	15360 (2)	
Number of BACs with WGP tags	44810	21735	66545
% BACs with WGP tags (st. dev.) **	91.3 (3.6)	71.1 (4.5)	
Average number of tags/BAC	38	26	34
Average number of reads/tag	63	65	57
Unique WGP tag sequences (26 bp)			322437

* Calculated from clone size and assuming a genome size of 850 Mb

** This count excludes 22 (partially) failed RHPOTKEY library plates

The WGP fingerprints were prepared for physical map construction with FPC by replacing the tag sequences by randomly chosen ID numbers, that serve as pseudo band mobility values for fingerprint alignment (Additional file 5). Chimeric WGP fingerprints were removed from the dataset and the remaining 65919 BACs, representing 9.0 g.e. of DNA, were aligned into a physical map. The map was built with a relatively relaxed alignment cut-off value of 1e-21. Contigs with more than 5 questionable clones were split up and re-aligned with the DQ-er function in three steps of increasing stringency (cut-off values 1e-24, 1e-27, and 1e-30). Finally two rounds of automated end-end merging were performed between the contigs at thresholds of 1e-21, and then 1e-18.

The resulting WGP physical map has a length of 1396 Mb, with 3601 contigs containing 53138 clones and 12781 single BACs (Table 1; Additional file 1). The WGP map shares 39733 RHPOTKEY clones with the AFLP physical map. As a result, nearly all AFLP marker anchor points could be carried over to the WGP map, and 1127 contigs were anchored. The BAC alignments in the physical map contigs result in a partial ordering of the WGP tags, which generates contig-wide sequence scaffolds that can be used for anchoring of WGS sequences (e.g. see Additional file 5).

It was anticipated that the use of the sheared RHPOTLUC library in the WGP map would close gaps between BAC contigs that cannot be bridged by the partial digest clones of the RHPOTKEY library. However, in the contigs of WGP physical map (and of the hybrid map below), very little evidence was found of gap closure by the sheared BACs. It is therefore concluded that the WGP physical map did not benefit from the incorporation of the sheared library clones. A possible explanation for this failure is that the gap-filling clones of the sheared library contain very repetitive sequences, and could therefore not be fingerprinted with the current WGP protocol. Possibly, the 20% lower yield of clones with WGP tags in the RHPOTLUC library is correlated with the failure to find gap-filling clones.

### Integration of AFLP and WGP physical maps into hybrid map

The 39733 clones that are shared by the AFLP and WGP physical maps were used to identify contig overlaps between both maps. A search was made for contig pairs from the AFLP and WGP map that had at least two BAC clones in common. The AFLP and WGP contigs that were connected via such a BAC link, or via a chain of such BAC links, were placed in contig groups. A total of 1167 contig groups were identified that contained one pair of contigs from both maps. In addition, 929 groups were identified that joined three or more contigs from both maps. Within each physical map, the number of contigs was recounted, with each contig group now being taken as a single contig. This group enhancement brought the number of contigs in the AFLP map down by 32% to 2819 and in the WGP map down by 22% to 2785 (Table 1). Additional file 6 shows an example of contig grouping between both maps. The contig descriptions of both physical maps, with their contig grouping information, have been combined in a single table that describes the hybrid potato physical map (Additional file 1).

### Comparison of AFLP and WGP physical maps

The map integration via contig groups showed that nearly all larger contigs were mirrored in both maps. The only notable difference was that the AFLP map contained a 96-clone contig from the NOR (Figure 5), and two other contigs of 28 and 41 clones with uniform, simple fingerprints, that were not seen in the WGP physical map. This difference most likely is caused by a high content of repetitive sequences in these BACs, since such sequence tags will fail to resolve with the WGP sequencing protocol. Similarly, the chloroplast fingerprints of the AFLP map were absent in the WGP map, because their presence in 14-15 clones per library plate will prevent deconvolution of their WGP sequence tags.

The contig build in the WGP map was of a better quality than that of the AFLP map (Table 1). Using less fingerprints (53138 versus 59747), the number of contigs was smaller (3601 versus 4150) and yet the genome coverage by the total contig length was slightly better than the AFLP map (1396 versus 1361 Mb). This difference in quality is also reflected in both the average and N50 contig sizes (Table 1).

In terms of map construction, the WGP physical map had more difficulty than the AFLP map with removing friction in the BAC alignments, which is reflected in the higher number of contigs with five or more questionable clones (Table 1). Chimeric fingerprints gave more severe disturbances of the BAC alignments in the WGP map, as compared to the AFLP map, and it was necessary to remove them as much as possible. Well-to-well fingerprint contaminations, on the other hand, were present in the AFLP contigs, but absent in the WGP contigs.

The WGP physical map has a much higher proportion (19.4%) of singleton clones than the AFLP map (7.3%) (Table 1). One explanation for this difference is that the AFLP physical map did not include clones with less than 10 bands, which may have kept its singleton count low. However, a better explanation is found in the different shapes of the fingerprint band distributions of both maps (Figures 2 and 7). In the WGP map, the distribution of the number of tags per BAC is asymmetric compared to the fingerprint band distribution of the AFLP map. The WGP tag distribution maintains a very wide tail for BACs that have less than 15 tags. A likely explanation for this overrepresentation of WGP fingerprints in the low end of the distribution is that they belong to clones of normal length that are missing relatively many of their sequence tags, because these are repetitive sequence tags, which are not resolved by the WGP sequencing [25]. Such sparsely tagged BACs are more likely to remain singleton clones in the physical map, which fits the sharp rise in singleton clones towards the low end of the distribution.

### Heterozygosity analysis

The AFLP markers of the potato physical map are heterozygous markers that specifically identify BAC clones that belong to either the phase {0} haplotype or the phase {1} haplotype of their chromosome (see for example Additional file 6). Although an AFLP marker locus can have two allelic bands of the opposite haplotype, such allele pairs are not revealed in the potato genetic map, and the AFLP markers are treated as presence/absence (i.e. dominant) markers with only a single allele. This means that the potato AFLP markers cannot be used for the evaluation of physical map heterozygosity through direct identification of allelic BAC clones, as was done with codominant SNP markers in the grape physical map [20].

Nevertheless, indirect evidence that genome heterozygosity has resulted in haplotype-specific BAC fingerprint alignments in both physical maps can be derived from the distribution of the AFLP marker haplotypes in the BAC contigs (Table 4). For the 405 BAC contigs with two or more AFLP markers in the AFLP physical map, it was examined what their haplotype composition is. When multiple AFLP markers are present in the contigs, there is a strong preference for the markers to stay within one haplotype, and the observed percentages of two-haplotype contigs systematically were much lower than what would be expected on the basis of an independent combination of marker haplotypes (Table 4). The ultradense genetic map of clone RH [9] shows that markers of both haplotypes occur mixed throughout the genome, and that there is little or no haplotype preference in different genetic regions. Therefore, the clear avoidance of AFLP marker haplotype mixing in the anchored physical map contigs suggests that their BAC clones are to a large degree haplotype specific. Those contigs that do combine AFLP markers of two haplotypes are on average longer than the single haplotype contigs with the same number of markers (Table 4). This increased length suggests that incorporation of fingerprints of a second haplotype in a contig does not lead to their full integration, but will instead keep them as a haplotype-specific segment within the contig. Similar results were found when analysing the marker haplotype distribution in the 423 multi-anchor contigs of the WGP physical map (data not shown). The only notable difference compared to the AFLP physical map was that mixed-haplotype contigs were slightly more abundant among the 2-marker contigs (20% mixed) and among the 3-marker contigs (35% mixed).

**Table 4:** Marker haplotype distribution in contigs of the AFLP physical map

AFLP markers per contig	One haplotype in contig *		Two haplotypes in contig **		% Two-haplotype contigs	
	
	# Contigs	Avg. kb***	# Contigs	Avg. kb***	Observed	Expected ****
1	847	378				
2	225	469	32	682	12.5	50
3	75	609	14	910	15.7	75
4	26	617	14	871	35.0	87.5
5	9	808	6	1078	40.0	93.8
6	2	687	1	810	33.3	96.9
7	0	0	2	1732	100.0	98.5

* The AFLP markers that anchor the contig belong to the same haplotype

** The AFLP markers that anchor the contig come from both haplotypes of the genome

*** Average length of the contigs (in kb) estimated from the number of fingerprint bands

**** Expected value when AFLP marker haplotypes combine independently in the BAC contigs

**Figure 8:**
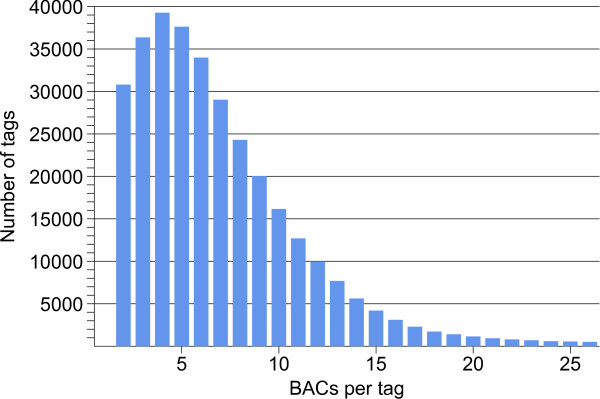
**Frequency distribution of WGP sequence tags in 66545 BACs of potato clone RH**. Sequence tags that were present in only one BAC clone were not included in the WGP dataset.

**Figure 9:**
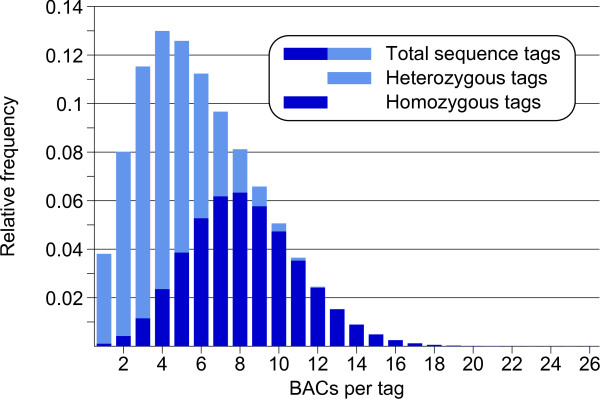
**Theoretical frequency distribution of WGP sequence tags**. This distribution is based on 8.2 genome equivalents of template DNA and 54% of heterozygous tags, and is a combination of two Poisson distributions for respectively the heterozygous and homozygous tags. It gives a good fit to the lower half of the observed distribution (Figure 8) and accommodates the relatively high fraction of two-copy tags in the WGP-dataset. However, other distributions with slightly higher g.e. and heterozygosity values will fit as well. The theoretical distribution assumes that all sequence tags are derived from single loci, and that no losses or errors have occurred with WGP sequencing. The relatively thick tail in the observed distribution (Figure 8) indicates that some of the tag sequences are likely to have come from duplicated loci.

The heterozyogous nature of the RH genome is also revealed by the frequency distribution of the WGP sequence tags (Figure 8). This distribution has a maximum at 4 BACs per tag, which is much less than would be expected from the estimated 9 genome equivalents of BAC DNA that produced the tags (Table 3). The shape of the observed distribution can be explained as being composed of two separate frequency distributions for respectively heterozygous and homozygous sequence tags. A good approximation of the observed distribution is given by the theoretical distribution for 8.2 genome equivalents of template DNA, in which the ratio of heterozygous to homozygous tags is set to be 1.2 to 1 (Figure 9). This interpretation of the frequency distribution would mean that roughly 54% of the WGP tags are heterozygous. Such a high level of heterozygosity will be reflected in the alignment of the BAC WGP fingerprints, and is likely to have favoured the formation of haplotype-specific contigs in the WGP physical map.

### Map inflation

Both the AFLP and WGP physical map presented a total contig length of approximately 1396 Mb (Table 1). This physical map length is 1.64 times larger than the 850 Mb size of the haploid potato genome. Such a high level of map inflation was also found for two physical maps of grape [18,20], where it was attributed largely to the heterozygous nature of the grape genome. Heterozygosity-induced assembly of haplotype-specific BAC contigs causes genomic regions to occur twice in a diploid physical map, resulting in an increase of the total map length. Both the AFLP marker haplotype distribution in the potato physical map and the WGP sequence tag copy number distribution have provided indirect evidence of heterozygosity among the potato BAC fingerprints, and these observations provide an explanation the large length inflation of the two potato physical maps.

The potato physical maps were constructed by automated calculations with the FPC software, without further manual merging of contigs. Even though the final contig merges were carried through at relaxed alignment settings, it is likely that undetected contig overlaps still exist in both potato physical maps. Such undetected contig overlaps contributed, for instance, to the 1.26 fold length inflation of the soybean physical map [37], and will therefore in part also explain the inflation of our potato physical maps.

## Conclusions

Presented here are the first two genome-wide BAC physical maps of potato, which come from the heterozygous diploid genotype RH89-039-16. These maps serve as an important resource and reference framework for current and future potato research. In this paper we have reported the strategies by which these maps have been made, as well as detailed statistics about them. We have also compared our methods with other recently constructed physical maps.

The AFLP physical map is genetically linked to the ultradense genetic map of genotype RH [9]. A distinctive feature of this genetic map, as opposed to e.g. EST-based genetic maps, is that it has a large proportion of its markers in the pericentromeric, low recombination regions of the chromosomes. This means that the potato physical map is unique in providing an extensive genetic handle on the BAC sequences from these heterochromatic regions.

The combined potato physical maps provide a high resolution genome-wide scaffold structure composed of WGP sequence tags and BAC end sequences [38], which can be used for anchoring and ordering of whole genome and BAC pool derived shotgun sequence assemblies. With 10% of the heterozygous RH genome having been sequenced on a traditional BAC by BAC basis [29], the sequence scaffolds of the physical map can facilitate the sequencing of the remainder of the RH genome by more high throughput methods.

The physical maps as they are presented here were optimized for having a low number of contigs, and these contigs illustrate the level of fingerprint connectivity that is available in the physical maps between the BACs of a given genomic region. However, this optimization is likely to have caused local misalignments of BACs in contigs with mixed haplotypes. For a proper assessment of BAC order and for an evaluation of the effect of heterozygosity on BAC fingerprint alignments it will be necessary to compare the physical map contigs with potato sequence data.

Of particular interest is the recent finishing of the genome sequence of a doubled monoploid potato (DM) of the *Solanum tuberosum *group *phureja *by a WGS approach [29]. The RH physical map can be aligned to this homozygous potato sequence via the sequences of the WGP tags and of the BAC ends. The integration of these two genomics resources will set a new baseline for molecular research in potato that will enable cross-genome gene comparisons between the three haplotypes in genotypes RH and DM. Also, the combined framework will serve as a reference onto which sequences from other potato genotypes, including tetraploids, can be placed. These new possibilities will advance functional genomics studies in potato and also stimulate the breeding of potato varieties with novel or improved quality and agronomic traits.

## Methods

### BAC libraries

Genomic DNA of the diploid potato genotype RH89-039-16 [9] was used to construct two BAC libraries for physical map fingerprinting. The RHPOTKEY library consists of 78336 clones in the vector pIndigoBAC5 in *Escherichia coli *DH5-alpha, and was made by KeyGene N.V. (Wageningen, The Netherlands) by partial digestion with either HindIII (45696 clones; plates 1-119) or EcoRI (32640 clones; plates 120-204). With an average clone size of 127 kb (st. dev. 37 kb; n = 590) this library has a coverage of 11.7 genome equivalents. In the physical map, the RHPOTKEY clones have names beginning with "RH". The RHPOTKEY library has been end-sequenced [38].

The RHPOTLUC library was produced by the Lucigen Corporation (Middleton, WI) from sheared DNA, which was cloned into the vector pSMART-BAC, and transformed into BAC-Optimized replicator *E. coli *cells. The library has 85248 clones with an average size of 96 kb (st. dev. 34 kb; n = 131), and an estimated coverage of 9.5 g.e. In the physical map, RHPOTLUC clones have names beginning with "PL".

A third 35712-clone HindIII BAC library of genotype RH was made at the James Hutton Institute (Dundee, UK). This library has been used for targeted AFLP marker screening [39] and a few selected clones have been AFLP-fingerprinted and incorporated in the physical map. These BAC clones were provided by Dr. Glenn Bryan and were given names starting with "GB".

### BAC AFLP fingerprinting

By sampling each 384-well library plate four times with a 96-pin replicator, the BACs from the RHPOTKEY library were grown in 1.5 ml of Terrific Broth in deep 96-well blocks sealed with AirPore tape [40]. BAC DNA was isolated from these cultures with a standard alkaline lysis miniprep, and 300 μl of the cleared lysate was transferred to a new deepwell plate for isopropanol precipitation of the BAC DNAs. Following EcoRI/MseI restriction and AFLP adapter ligation, the BAC DNA samples were subjected to AFLP PCR in 96-well plates, using EcoRI and MseI AFLP primers without selective nucleotides [21]. This so-called non-selective (or +0/+0) AFLP reaction will amplify all EcoRI/MseI fragments from the BAC DNA, including AFLP markers (if any) from the genetic map. For each PCR plate, the EcoRI primer was labelled with one of the three fluorescent dyes FAM, JOE or NED. Samples from three PCR plates with different dyes were combined, supplemented with ET-ROX labelled size ladder and separated by electrophoresis in a 96-capillary MegaBACE 1000 sequencer (Amersham Biosciences) at KeyGene N.V. (Wageningen, The Netherlands). Using proprietary band-calling software (BACXtractor), AFLP bands were sized and scored from the fluorescent trace files, and the mobilities of the AFLP bands and the heights of their fluorescence peaks were saved in a two-column text file format ('extended bands file format') that is compatible with the BAC alignment software FPC [10,13]. Capillary fingerprinting will size AFLP bands as decimal numbers in the 60-900 bp range, with a sizing accuracy of about 0.3 bp. However, these high-resolution fingerprint data cannot be used directly by the FPC software because it only accepts 16-bit whole numbers as band mobility values, and would round off the mobilities to the nearest whole base pair. To avoid this loss of data accuracy, the capillary band mobilities were multiplied by a factor 10, which enables accurate alignment by FPC. The extended bands file format (Figure 1B) was used for viewing and evaluating the fingerprints with custom-written software. For alignment with FPC, the peak height values are discarded and only the band mobilities are used. One of the two MegaBACE machines that were in use for BAC fingerprinting gave systematic sizing errors. A band size correction was applied to the affected fingerprints, which was based on calibration information derived from the omnipresent chloroplast DNA fingerprints in the dataset. More details about the fingerprint procedure can be found in reference [34].

### AFLP physical map construction

Preliminary versions of the AFLP physical map were constructed with FPC V6.4. In these calculations, which used the equation 2 alignment algorithm, the tolerance and cut-off parameters were varied independently to determine their optimum values. An optimal physical map quality, with relatively large contigs containing few questionable clones was obtained with a band size tolerance of 5 (which corresponds to a 0.5 bp size difference in the fingerprints) and an alignment cut-off value of 1e-11. The physical map created with these optimal settings was used for a first analysis of the data. This map revealed a single large contig (~2700 BACs) of chloroplast DNA-derived clones, of which some BACs, such as RH180I06, contained the complete potato cpDNA. Also, it revealed a contaminating artefact band pattern that affected about 4% of the fingerprints, and which presumably is of *E. coli *origin. Also these artefact fingerprints aggregated into a single large contig. The final version of the AFLP map was made with FPC V9.3 [13], which has a neighbouring well contamination search option that was used to identify an additional 4.4% of (potentially) contaminated or mixed fingerprints. In the final AFLP map all contaminating fingerprints were removed.

The final map was built with a more relaxed alignment cut-off value of 1e-09, followed by removal of entanglements with the DQ-er function at cut-offs of 1e-10 to 1e-12. With this setting, a maximum incorporation of clones was achieved, while questionable clone alignments could still be nearly completely removed. The number of contigs then was reduced by automated end-to-end merging with standard settings (minimal 2 clones overlap) at 1e-08 and 1e-07. Further contig merges at 1e-06 or higher were not performed, because they began to lead to false contig links, as indicated by conflicts in the AFLP marker anchor points. Genome coverage was estimated from an average size of 130 kb per insert-containing RHPOTKEY BAC clone and an average of 37.38 bands per fingerprint in the final physical map, which gives 3477 bp of sequence per fingerprint band in the physical map. This parameter was used to calculate all contig length statistics of the AFLP physical map. With a total of 391465 aligned bands in all contigs, this gives an AFLP physical map length of 1361 Mb.

### BAC library pooling

A unique and efficient pooling strategy has been applied to the RHPOTKEY BAC library in order to screen it for AFLP markers of the ultradense genetic map. The aim was to locate each copy of a marker in the library within an accuracy of a quarter library plate segment of 96 BAC clones. To this end, 764 pooled DNA samples were prepared from the quarter segments of 191 (of the 204) 384-well library plates. These quarter plate pool (QPP) DNA samples then were used as the pooling units in a random k-sets pooling design, with k = 4 and v = 90 and n = 764, as outlined by Bruno *et al*. [27] for single BACs. The result is a set of 90 DNA superpools from which the genetic marker scores can be deconvoluted into a series of positive QPPs, effectively screening 764 QPP DNA samples in a single pass.

The QPP samples were prepared by pooling the leftover cleared lysates from the 96-well BAC DNA isolations of the AFLP physical map. Typically, 20 ml of pooled lysate was collected per 96-well block. The QPP BAC DNA was pelleted by isopropanol precipitation and dissolved in 600 μl of Tris-EDTA buffer (pH 8). The ninety DNA superpools were prepared by manually pipetting each QPP DNA sample into a unique set of four superpool samples (using 20 μl QPP DNA per superpool), according to the random k-sets pooling design. Track was kept of a small number of pipetting errors, which were taken up the description and deconvolution of the pooling design. The QPPs were distributed pseudorandomly across the superpools, with small corrections so that each superpool contained 33 or 34 QPP samples. Each superpool sample corresponds to approximately 0.44 genome equivalents of potato DNA, which gives AFLP patterns with a complexity and appearance that come close to the AFLP patterns from the complete genomic DNA of genotype RH.

### Characteristics of the BAC pooling design

**Figure 10:**
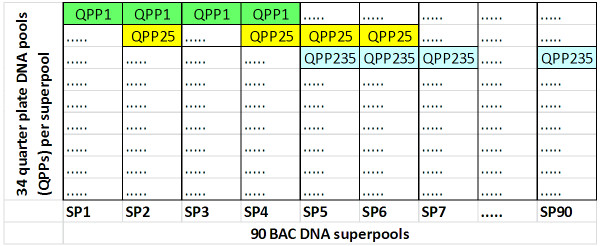
**Principle of the DNA superpool design of the RHPOTKEY BAC library**. Pooled DNAs from 764 quarter library plates (quarter plate pools, QPPs) are each added to a unique combination of four different superpools (SP1...SP90), as shown here for three QPPs. Genetic marker screening is performed on the 90 superpools, and the marker-positive QPPs are then identified by deconvolution of the pooling design (see text for explanation).

The principle of the potato random k-sets BAC pooling design is illustrated with a fictitious example in Figure 10. An AFLP marker that is present in one of the 96 BACs of quarter plate pool QPP1 will be visible in the AFLP pattern of superpools SP1 to SP4. In reverse, if a marker is present in SP1 to SP4, then it must come from a BAC in QPP1, since this is the only QPP that is present in all of these four superpools. A partial overlap in superpools between QPPs is allowed for deconvolution. For instance, if superpools SP1 to SP6 are positive for a marker, then this marker can still be assigned to both QPP1 and QPP25, because these are the only two QPPs that fall completely within this set of superpools. When the copy number of a marker increases in the BAC library, accidental overlaps begin to appear between QPP k-sets in the positive superpools, and the deconvolution of the positive QPPs will begin to obscure, with false positive QPPs appearing in the list of candidate QPPs. For instance, if SP1 to SP7 and SP90 are positive for a marker, then QPP1, QPP25 and QPP235 will be the output of the deconvolution of the pooling design since these QPP all fit in this superpool score. However, in this case the status of QPP25 is not clear. It is not needed to explain the superpool scores, and may therefore have or not have the marker. QPP1 and QPP235 are called resolved positive QPPs, because they are needed to explain the superpool scores and are therefore certain to contain the marker. On the other hand, QPP25 can be either a true positive QPP that remains unresolved, or a false positive QPP that it is present in all positive superpools by coincidence.

**Figure 11:**
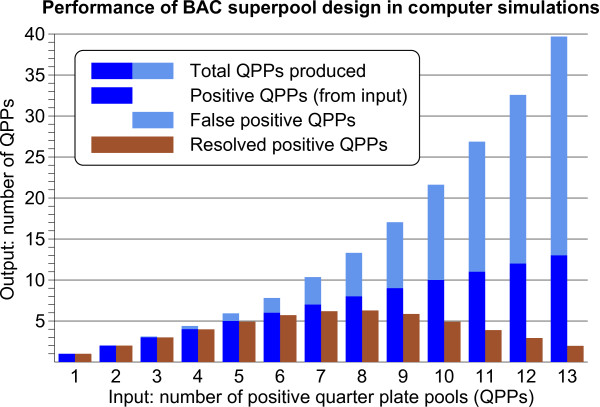
**Computer simulation of the behaviour of the BAC superpool design**. Shown is the deconvolution output, in terms of QPP categories, for different input numbers of marker-positive QPPs in the pooling design. Resolved positive QPPs are input QPPs that are recognized with certainty by the pooling design as being positive. At high input numbers of positive QPPs, not all of them can be resolved as being positive anymore and, in addition, false positive QPPs begin to contaminate the output list.

The theoretical performance of the BAC superpool design was evaluated with computer simulations (Figure 11) in order to obtain a reference standard by which the actual performance of the marker screening can be evaluated. Markers with BAC pool copy numbers varying from 2 to 13 were simulated by randomly choosing combinations of n positive QPPs (n = 2 to n = 13). With 1000 repetitions per n value, the positive superpools were calculated for each combination of QPPs. These positive superpool scores then were deconvoluted back to output lists with candidate QPPs, in which the resolved positive, unresolved positive and false positive QPPs were distinguished and their average counts were collected.

The results of the simulations (Figure 11) showed that up to an input of six positive QPP, these are accurately identified by the output list as resolved positives. However, as the number of positive input QPPs increases further, these can no longer be resolved completely, and the number of resolved positive QPPs actually declines. As a consequence, an increasing fraction of the positive QPPs is no longer recognized as such, and blends in with an increasing number of false positive QPPs. This "collapse" in resolving capacity at high marker copy numbers is a characteristic of k-sets pooling designs [27] and is a critical parameter in their use for BAC library screening per se. However, for the BAC anchoring procedure of the potato physical map this collapse of the pooling design was not an issue, because it compares the list of output QPPs with physical map data, and is thus able to identify the true positive QPPs, irrespective of the presence of false positive QPPs in the list.

### Marker identification and size conversion

RH-specific markers and bridge markers from 135 selective +3/+3 EcoRI/MseI AFLP primer combinations of the potato genetic map were traced back in the original autoradiogram films by looking for their segregation pattern in the 130 progeny lanes. This re-examination of gels corrected remaining deficiencies and mistakes in the radioactive marker sizing and had the added benefit of discovering 725 new markers for BAC anchoring. Radioactive AFLP gels for marker size conversion were prepared by KeyGene N.V. from DNA of 21 of the BAC superpools plus both parents of the genetic map, using these 135 primer combinations. Gels were prepared as described by Isidore *et al*. [41], but with the difference that the AFLP patterns were digitally captured by phosphor imaging. AFLP bands in the autoradiograms were sized with Improve software (KeyGene N.V., Wageningen) and further analysis of the raw image files was done with ImageJ software http://rsbweb.nih.gov/ij/.

### AFLP marker screening

Capillary AFLP patterns of the complete set of 90 BAC superpools plus the parents SH and RH of the genetic map were made with the 135 selective +3/+3 EcoRI/MseI primer combinations by KeyGene N.V. (Wageningen, The Netherlands) essentially as described for the BAC fingerprinting. Because the NED dye gave weak AFLP patterns, only two PCR plates with AFLP samples (labelled with FAM and JOE) were combined within a MegaBACE run. Capillary fingerprint patterns were called with BACXtractor software (KeyGene N.V., Wageningen) and saved as extended bands files as described for the BACs. Custom software was written to do all analyses on these BAC pool bands files. AFLP markers were identified in the capillary bands files by visual pattern comparison with the radioactive BAC pool gels. A size interval was determined (typically 0.2 to 0.4 bp wide) that spanned the marker band in the BAC pool bands files, and from the bands in this interval the average marker size was calculated. If a marker could not be identified with reasonable confidence, e.g. because of interference with a neighbouring band, it was not used for anchoring. Absence of a marker in the BAC pools was another cause of losing anchor markers.

### *In silico *anchoring of BAC contigs

For each primer combination, the BAC superpools having AFLP marker bands were identified by automated scoring of the capillary bands files within the pre-set marker size intervals, and the data were saved in separate superpool score files for each marker. These score files were run through a software script that deconvolutes the superpool design, producing a list of candidate positive quarter plate pool IDs for each AFLP marker. A second script was then used to compare the BAC contigs of the physical map against the positive QPP of a marker. By choosing an appropriate threshold of a minimum number clones in a contig that have to be present in the QPPs, a short list of matching BAC contigs was produced, which displayed the BAC clones having a fingerprint band within 0.4 bp distance of the AFLP marker size. The contig with the marker band then was identified by eye from the short list and the marker-positive BAC names were taken up in a database with anchoring results. Whenever a contig showed less positive clones than was expected on the basis of the number of QPPs, an overlapping contig (or singleton BAC) was sought for with FPC, and any additional marker-positive clones in this overlapping contig were added to the anchors database. The *in silico *search was often quite straightforward, finding a single matching contig without ambiguities, and would in many cases also have identified the contig without consulting the BAC fingerprints for the marker band. Although the BAC fingerprint bands of less than 100 bp were not used for the physical map construction, the AFLP markers below 100 bp were included in the anchoring and were identified in the unclipped BAC fingerprints.

### WGP physical map construction

Whole genome profiling sequence tags were purchased from KeyGene N.V. (Wageningen, The Netherlands) for 144 plates of the RHPOTKEY BAC library and for 80 plates of the RHPOTLUC library. The sequence tags were produced by high throughput sequencing of the EcoRI ends of non-selective AFLP fragments from BAC DNA pools [25]. To enable physical map construction with the publicly available FPC V9.3 software http://www.agcol.arizona.edu/software/fpc/, the 322234 unique tag sequences in the WGP dataset were converted to pseudo band mobility values, by randomly assigning ID numbers in the range 1000-54705 (i.e. within the 16-bit length used by FPC) to each tag sequence, with each ID number being given out to six tag sequences. For each BAC, a pseudo bands file was then created by replacing the tag sequences by their mobility number, and these pseudo bands files then were imported into FPC. The WGP fingerprints were cleaned from chimeras by looking for BACs that gave false connections or friction alignments in preliminary versions of the physical map, and also by looking for BACs with chimeric WGP tag alignments to a pre-publication version of the *Solanum tuberosum *group *phureja *genome sequence [29]. The WGP physical map was built with the equation 2 algorithm, using a band size tolerance value of 0, which specifies to the FPC software that only exact matches between sequence tag ID numbers are valid for fingerprint alignment. The cut-off probability was set to 1e-21. At higher cut-off values, false connections began to appear in the build, which were recognized by their conflicting anchoring information. These false connections were supported by more than one fingerprint (i.e. were not caused by chimeras) and were therefore seen as unwanted accidental fingerprint similarities that were surfacing at these higher cut-off settings. The removal of questionable (Qs) clones was difficult in the WGP map. Large DQ-er cut-off steps of 1e-24, 1e-27 and 1e-30 were needed to split 75% of the 304 contigs with 5 or more Qs clones, and the remaining more persistent Qs contigs were left as they were. Automated contig end to end merging at 1e-18, requiring two BACs to confirm the overlap, was then used to bring the contig count down from 3800 to 3600. At this 1e-18 stage, no false BAC connections were observed, as indicated by the AFLP marker anchor points. The average sequence coverage per WGP tag was estimated from the RHPOTKEY BACs only, because their pulsed-field sizing data were considered to be more accurate than the sizing of the RHPOTLUC BACs, and also because this gives a more accurate comparison with the AFLP physical map. With an average of 37.37 WGP tags per RHPOTKEY clone in the cleaned fingerprint set, this translates into 3477 bp of sequence per WGP tag. This parameter was used to calculate all contig length statistics of the WGP physical map. With a total of 401465 aligned bands in all contigs, this gives a WGP physical map length of 1396 Mb.

### Probability calculations haplotype mixing

If allelic BAC fingerprints from both diploid haplotypes can combine freely in the contigs of the physical map, and if the haplotypes ({0} or {1}) of the available AFLP anchor markers for a given genomic region are determined by chance, then a probability can be calculated that a multi-anchor contig will have AFLP markers of only a single haplotype. For two-marker contigs, the probability that both markers are of the same haplotype is 0.5. For contigs with n AFLP markers, this value becomes 0.5^(n-1)^. The alternative probability that an n-marker contig will have markers of two haplotypes then becomes 1- 0.5^(n-1)^. Using this latter probability, which applies when heterozygosity does not affect the alignment of the BAC fingerprints, the expected percentage of two-haplotype contigs was calculated separately for each class (n = 2 to n = 7) of multi-anchor contigs.

## List of abbreviations

AFLP: DNA fingerprinting technique that detects genomic restriction fragments; Avg.: Average; BAC: Bacterial Artificial Chromosome; cpDNA: Chloroplast DNA; DQer: Function in FPC that Decreases the number of Qs clones; EST: Expressed Sequence Tag; ET-ROX: Rhodamine reporter dye, excited through energy transfer by FAM; FAM: 6-Carboxyfluorescein; FISH: Fluorescence In Situ Hybridization; FPC: Finger Printed Contigs, the name of the software for fingerprint alignment; g.e.: Haploid genome equivalents; JOE: 5'-Dichloro-dimethoxy-fluorescein; kb: Kilo base pairs; KeyMaps: AFLP anchoring procedure for physical maps; Mb: Mega base pairs; N50: Value position in a sorted list of values at which the sum of the preceding values has reached 50% of the total sum of the values; NED: Proprietary fluorescent dye from Life Technologies; NOR: Nucleolar Organizer Region; QPP: Quarter (library) Plate (DNA) Pool; PCR: Polymerase Chain Reaction; PL: Name prefix of BAC clones from the RHPOTLUC library; Qs: Questionable, a status given by FPC to poorly aligned BAC clones in a contig; RFLP: Restriction Fragment Length Polymorphism; RH: diploid potato clone RH89-039-16 and name prefix of BAC clones from the RHPOTKEY library; RHPOTKEY: Name of BAC library, RH POTato KEYgene; RHPOTLUC: Name of BAC library, RH POTato LUCigen; SNP: Single Nucleotide Polymorphism; SSR: Simple Sequence Repeat; st. dev.: Standard deviation; WGP: Whole Genome Profiling; WGS: Whole Genome Shotgun.

## Authors' contributions

JMdB performed band calling of AFLP fingerprints, processed AFLP and WGP fingerprint data, constructed AFLP and WGP physical maps, supervised and performed AFLP marker localisation in radioactive gel patterns of the genetic map, performed AFLP marker size conversion and anchoring, analysed and curated physical map data, isolated BAC DNA and prepared BAC quarter plate pool DNAs, wrote software for data processing and analysis, and drafted the manuscript. TJAB characterized and improved BAC AFLP fingerprints, constructed AFLP physical maps, analysed AFLP physical map data, contributed to AFLP marker anchoring, isolated BAC DNA, designed and prepared BAC DNA superpools, wrote software for analysis and presentation of physical map data and provided critical comments on the manuscript. TJ supervised RHPOTKEY BAC library construction and AFLP BAC fingerprinting, conceived and developed the KeyMaps anchoring procedure and provided critical comments on the manuscript. BB set up and performed the BAC fingerprint AFLP reactions. XT performed the BAC FISH experiment to identify the NOR and performed AFLP anchor verifications with BAC FISH. GJB facilitated part of the data analysis work by JMdB at the J Hutton Institute, performed BAC library marker screening, contributed AFLP anchor BACs and provided critical comments on the manuscript. JB conceived and wrote the APOPHYS physical map project proposal. HJvE was involved in project writing, provided support for genetic map-related issues, and gave critical comments on the manuscript. RGFV was involved in project writing and funding acquisition, supervised the physical map project and provided critical input on composing the manuscript. All authors read and approved the final manuscript.

## Supplementary Material

Additional file 1 **Integrated hybrid AFLP and WGP physical map**. A table with all BACs incorporated in the potato AFLP and WGP physical maps, with their FPC contig numbers and contig alignment coordinates. Includes the contig group numbers of the hybrid physical map, which show the overlaps between contigs of both physical maps. Click here for file

Additional file 2 **AFLP marker size conversions**. list of AFLP marker mobilities in radioactive mapping gels versus capillary BAC fingerprint gels. Click here for file

Additional file 3 **List of AFLP anchor points**. A list of AFLP marker anchor locations in BACs of the AFLP physical map. Click here for file

Additional file 4 **Additional AFLP marker information**. Full names and genetic map information of AFLP markers in the AFLP physical map. Click here for file

Additional file 5 **Figure S1. Example of BAC alignment with WGP sequence tags in the WGP physical map**. Pseudo mobility values (ID numbers) were assigned to the 46 sequence tags of clone RH003F10 (left), which was then aligned into WGP physical map contig #3520 (right) on the basis of shared sequence tags with overlapping BAC clones. The consensus band map (CB map) shows the position of the sequence tags (red box) of BAC RH003F10 (highlighted in blue) relative to the neighboring clones. Plus signs indicate in which BAC clones the tags are present. On the basis of the BAC overlaps, a partial ordering of the sequence tags has taken place across the contig and a sequence scaffold is created that can be used for alignment of genomic sequences. Click here for file

Additional file 6 **Figure S2. Example of the integration of the AFLP and WGP physical maps of potato**. Contigs from the WGP physical map (red frames above) are matched with contigs from the AFLP physical map (blue frames below) on the basis of BAC clones that are present in both maps, as partially indicated by green connecting lines. AFLP contig #37 is connecting the four WGP contigs, and WGP contig #3076 connects the two AFLP contigs. Clone order is largely the same in both maps, but small deviations can be noticed. Parts of AFLP contig #37 are anchored to the two different haplotypes of chromosome 12 by AFLP markers that are in repulsion in the genetic map: blue squares indicate clones anchored to genetic haplotype {0} and pink squares mark clones from genetic haplotype {1}. Click here for file
