# Correction: A hybrid BAC physical map of potato: a framework for sequencing a heterozygous genome

**DOI:** 10.1186/1471-2164-13-423

**Published:** 2012-08-24

**Authors:** Jan M de Boer, Theo JA Borm, Taco Jesse, Bart Brugmans, Lian Wiggers-Perebolte, Linda de Leeuw, Xiaomin Tang, Glenn J Bryan, Jaap Bakker, Herman J van Eck, Richard GF Visser

**Affiliations:** 1 Wageningen UR Plant Breeding, Wageningen University and Research Centre, Droevendaalstesteeg 1, Wageningen, 6708 PD, The Netherlands; 2 Keygene N V, PO Box 216, Wageningen, 6700, The Netherlands; 3 The James Hutton Institute, Invergowrie, Dundee, Scotland, DD2 5DA, UK; 4 Laboratory of Nematology, Wageningen University, PO Box 8123, Wageningen, The Netherlands; 5 The Center for BioSystems Genomics, Wageningen, The Netherlands

## Correction

After the publication of the article ‘A hybrid BAC physical map of potato: a framework for sequencing a heterozygous genome. BMC Genomics 2011, 12:594’ [1] the submitting author was notified that the contributions of LWP and LdL were quite substantial. To properly show their contribution to the manuscript, they have been taken out of the acknowlegdements section and are now added to the authors list. LWP performed the BAC AFLP fingerprinting. LdL performed BAC pool AFLP reactions and BAC pool AFLP fingerprinting. Also, the submitting author was notified that patents and patent applications for technologies used in this publication should have been included in the acknowledgements section. Therefore, this article presents corrections to authors list, to the authors’ contributions section, and to the acknowledgements section. Also, we declare the existence competing interests. We apologize for any inconveniences these omissions may have caused.

### Corrected author list

Jan M. de Boer, Theo J.A. Borm, Taco Jesse, Bart Brugmans, Lian Wiggers-Perebolte, Linda de Leeuw, Xiaomin Tang, Glenn J. Bryan, Jaap Bakker, Herman J. van Eck, and Richard G.F. Visser.

## Competing interests

The authors declare that this publication has competing interests, due to the affiliation of authors TJ, LWP and LdL with the company Keygene N.V. in Wageningen. The AFLP®, KeyMaps and WGP™ technologies are covered by patents and patent applications owned by Keygene N.V. AFLP and WGP are (registered) trademarks owned by Keygene N.V.

## Corrected authors’ contributions

JMdB performed band calling of AFLP fingerprints, processed AFLP and WGP fingerprint data, constructed AFLP and WGP physical maps, supervised and performed AFLP marker localisation in radioactive gel patterns of the genetic map, performed AFLP marker size conversion and anchoring, analysed and curated physical map data, isolated BAC DNA and prepared BAC QPP pool DNAs, wrote software for data processing and analysis, and drafted the manuscript. TJAB characterized and improved BAC AFLP fingerprints, constructed AFLP physical maps, analysed AFLP physical map data, contributed to AFLP marker anchoring, isolated BAC DNA, designed and prepared BAC DNA superpools, wrote software for analysis and presentation of physical map data and provided critical comments on the manuscript. TJ supervised RHPOTKEY BAC library construction and AFLP BAC fingerprinting, conceived and developed the KeyMaps anchoring procedure and provided critical comments on the manuscript. BB performed BAC fingerprint AFLP reactions. LWP performed BAC AFLP fingerprinting. LdL performed BAC pool AFLP reactions and BAC pool AFLP fingerprinting. XT performed the BAC FISH experiment to identify the NOR and performed AFLP anchor verifications with BAC FISH. GJB facilitated part of the data analysis work by JMdB at the J Hutton Institute, performed BAC library marker screening, contributed AFLP anchor BACs and provided critical comments on the manuscript. JB conceived and wrote the APOPHYS physical map project proposal. HJvE was involved in project writing, provided support for genetic map-related issues, and gave critical comments on the manuscript. RGFV was involved in project writing and funding acquisition, supervised the physical map project and provided critical input on composing the manuscript. All authors read and approved the final manuscript.
