# Supplementary material for: A hybrid BAC physical map of potato: a framework for sequencing a heterozygous genome
Source: BMC Genomics. 2011 Dec 5;12:594. doi: 10.1186/1471-2164-12-594 (PMC3261212; doi:10.1186/1471-2164-12-594)

Sequence tags of BAC RH003F10	ID number
GAATTCTGATTTTCAAACATAAATAA	11343
GAATTCTATACGAAATCACGTTTGAT	6762
GAATTCTTTGTTTCCACAAGTTGGTT	9427
GAATTCAATAACAAAAGTGACTCCGT	13488
GAATTCCCAATCCACAGCGTGTGAGC	20539
GAATTCAGTTGCAGTGGCAGCCTCAG	25319
GAATTCAACGCAAATAACAAGTATGG	27609
GAATTCTGAGTTCGAGTTTTGAATAT	40225
GAATTCATGAACTAGTTTGGATTTCT	44352
GAATTCATATTGGAAAATTTCATGTT	7303
GAATTCCGAGTATATATTATGTTGTA	19745
GAATTCTAAAAGAATACTAATAATAA	23348
GAATTCTGTTGACCTAAATATTCAGA	30887
GAATTCCGATAAAGCAGACTCAAAAG	32360
GAATTCGTTCTGATAATTGATTATGT	36955
GAATTCTATTCGTTTCTATTAGAATC	41841
GAATTCTCAATGATGAAATCGACTAC	42562
GAATTCTTTCAAGTTTCTATGATACA	47501
GAATTCTCGACCTCCATACCCTTCTA	50651
GAATTCGGATAGTGAGTACAAAACAG	5203
GAATTCAGTTGCAGTGGCAGCCTCCG	45268
GAATTCTGATCAGAGCATAATGAACA	9066
GAATTCTGATTAGAGCATAATGAACA	23001
GAATTCATGTGTACTGTAGACATATA	32704
GAATTCATATATAAATTGTTTTAGTG	35872
GAATTCATGCCTGTCACCGGTAGGAC	8806
GAATTCTATGTTTAGTGCTTTGATTA	10060
GAATTCTTCTAATAACGCTCCAGCGC	12173
GAATTCCGTCCAAATATGAGTATCGT	14784
GAATTCTATTGATAAGGCAACAACTA	15106
GAATTCTTGAAAGTTACCTACAAAAG	23233
GAATTCACCATGACACTAGGATTATA	30628
GAATTCTACCACTCGCCGCTACTTCC	34890
GAATTCCTAGCGTGAACAAAGCAGAC	44696
GAATTCTTCCTCCAGAAACTAGGGAG	46120
GAATTCAACTTCAACCGCCGGCAAAC	48383
GAATTCTTCCAAATGGCCCACCCATT	50892
GAATTCTGATATTCAGAAATTGCTCT	9228
GAATTCTCTGAACAAAGCTTACCATT	49838
GAATTCTCTGCTGTAAATTGTATCTT	53638
GAATTCTAACAATTTCATTGATGTTA	21351
GAATTCCAACGCGTTGCTGGGATTGA	32380
GAATTCAAAATATGAAGAAGTAACAC	34814
GAATTCCACACAAGCTGATCAAGTTG	39450
GAATTCTAAGTGCAAATTCTGCTACT	41418
GAATTCAATTCCTCCAGATTCTTCGA	25131

Alignment in physical map

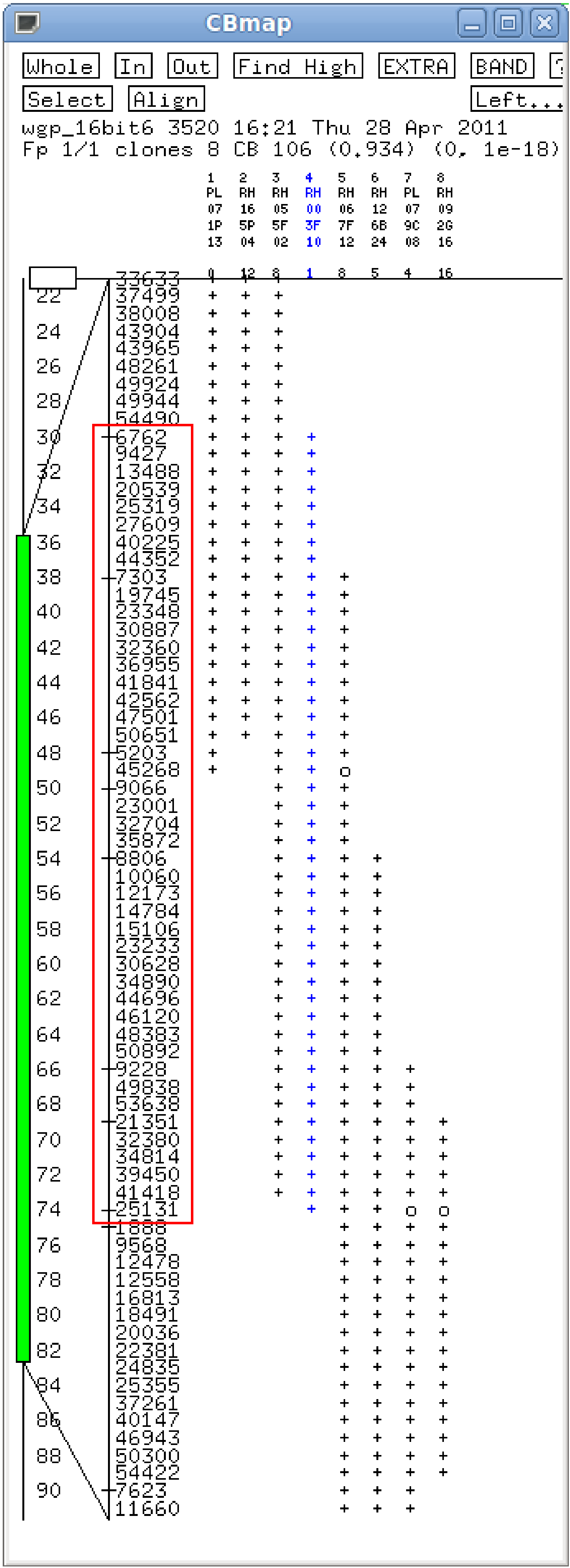

Supplement: Additional file 5 — Figure S1. Example of BAC alignment with WGP sequence tags in the WGP physical map. Pseudo mobility values (ID numbers) were assigned to the 46 sequence tags of clone RH003F10 (left), which was then aligned into WGP physical map contig #3520 (right) on the basis of shared sequence tags with overlapping BAC clones. The consensus band map (CB map) shows the position of the sequence tags (red box) of BAC RH003F10 (highlighted in blue) relative to the neighboring clones. Plus signs indicate in which BAC clones the tags are present. On the basis of the BAC overlaps, a partial ordering of the sequence tags has taken place across the contig and a sequence scaffold is created that can be used for alignment of genomic sequences. [file 1471-2164-12-594-S5.PDF]
