# Supplementary figures and images for: A hybrid BAC physical map of potato: a framework for sequencing a heterozygous genome
Source: BMC Genomics. 2011 Dec 5;12:594. doi: 10.1186/1471-2164-12-594 (PMC3261212; doi:10.1186/1471-2164-12-594)

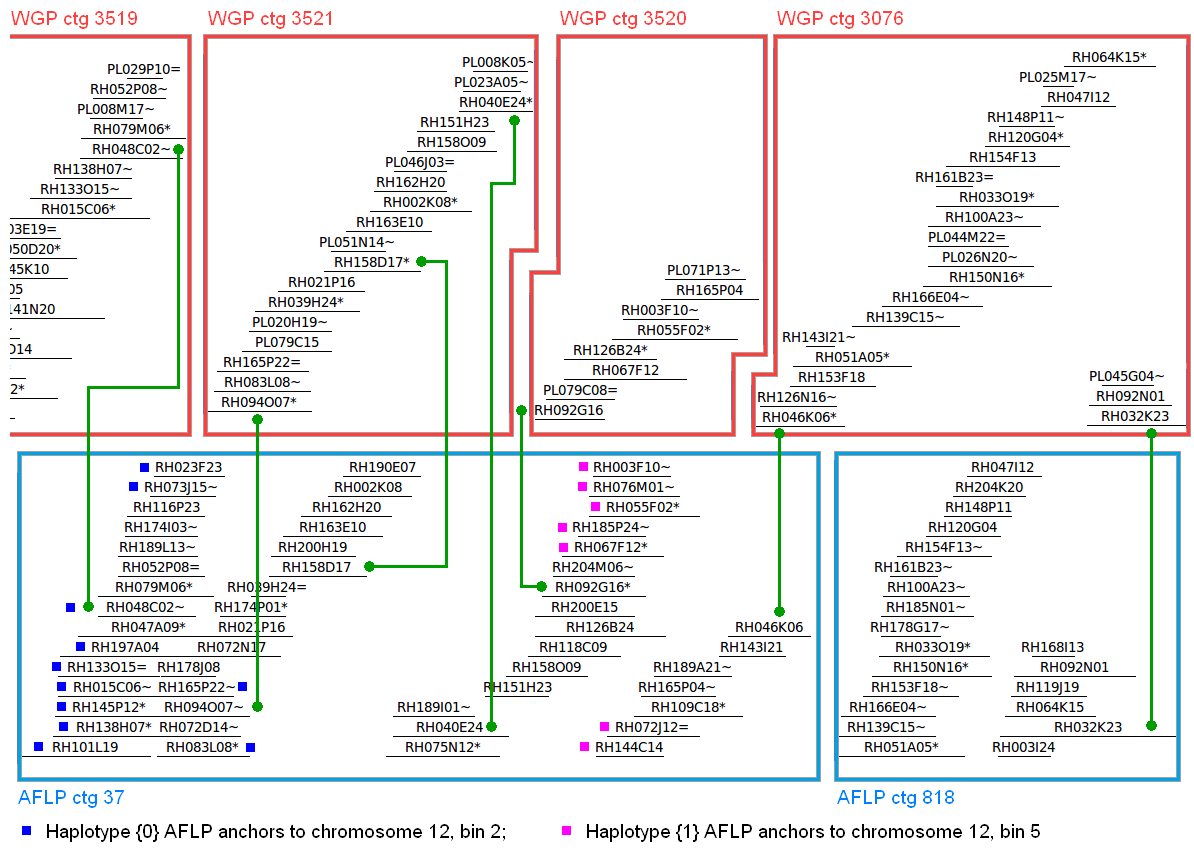

Supplement: Additional file 6 — Figure S2. Example of the integration of the AFLP and WGP physical maps of potato. Contigs from the WGP physical map (red frames above) are matched with contigs from the AFLP physical map (blue frames below) on the basis of BAC clones that are present in both maps, as partially indicated by green connecting lines. AFLP contig #37 is connecting the four WGP contigs, and WGP contig #3076 connects the two AFLP contigs. Clone order is largely the same in both maps, but small deviations can be noticed. Parts of AFLP contig #37 are anchored to the two different haplotypes of chromosome 12 by AFLP markers that are in repulsion in the genetic map: blue squares indicate clones anchored to genetic haplotype {0} and pink squares mark clones from genetic haplotype {1}. [file 1471-2164-12-594-S6.TIFF]
